# Biochemical activity induced by a germline variation in *KLK3* (PSA) associates with cellular function and clinical outcome in prostate cancer

**DOI:** 10.21203/rs.3.rs-2650312/v1

**Published:** 2023-03-28

**Authors:** Srilakshmi Srinivasan, Thomas Kryza, Nathalie Bock, Brian WC Tse, Kamil A. Sokolowski, Janaththani Panchadsaram, Leire Moya, Carson Stephens, Ying Dong, Joan Röhl, Saeid Alinezhad, Ian Vela, Joanna L. Perry-Keene, Katie Buzacott, The IMPACT Study, Manuela Gago-Dominguez, The PROFILE Study Steering Committee, Johanna Schleutker, Christiane Maier, Kenneth Muir, Catherine M. Tangen, Henrik Gronberg, Nora Pashayan, Demetrius Albanes, Alicja Wolk, Janet L. Stanford, Sonja I. Berndt, Lorelei A. Mucci, Stella Koutros, Olivier Cussenot, Karina Dalsgaard Sorensen, Eli Marie Grindedal, Timothy J. Key, Christopher A. Haiman, Graham G. Giles, Ana Vega, Fredrik Wiklund, David E. Neal, Manolis Kogevinas, Meir J. Stampfer, Børge G. Nordestgaard, Hermann Brenner, Marija Gamulin, Frank Claessens, Olle Melander, Anders Dahlin, Pär Stattin, Göran Hallmans, Christel Häggström, Robert Johansson, Elin Thysell, Ann-Charlotte Rönn, Weiqiang Li, Nigel Brown, Goce Dimeski, Benjamin Shepherd, Tokhir Dadaev, Mark N. Brook, Amanda B. Spurdle, Ulf-Håkan Stenman, Hannu Koistinen, Zsofia Kote-Jarai, Robert J. Klein, Hans Lilja, Rupert C. Ecker, Rosalind Eeles, The Practical Consortium, The Australian Prostate Cancer BioResource, Judith Clements, Jyotsna Batra

**Affiliations:** 1 School of Biomedical Sciences, Faculty of Health, Queensland University of Technology (QUT),; 2 Translational Research Institute, Queensland University of Technology, Woolloongabba, Brisbane, Queensland (QLD), Australia.; 3 Mater Research Institute - The University of Queensland, Translational Research Institute, Woolloongabba, Brisbane, QLD, Australia.; 4 Preclinical Imaging Facility, Translational Research Institute, Woolloongabba, Brisbane, QLD, Australia.; 5 Department of Urology, Princess Alexandra Hospital, Brisbane, Woolloongabba, Brisbane, QLD, Australia.; 6 Pathology Queensland, Sunshine Coast University Hospital Laboratory, Birtinya, Sunshine Coast, QLD, Australia.; 7 The Institute of Cancer Research, London, SM2 5NG, UK.; 8 Royal Marsden NHS Foundation Trust, London, UK.; 9 Genomic Medicine Group, Galician Foundation of Genomic Medicine, IDIS, Complejo Hospitalario Universitario de Santiago, SERGAS, Santiago de Compostela, Spain.; 10 Ronald and Rita McAulay Foundation, London, UK.; 11 Centre for Cancer Genetic Epidemiology, University of Cambridge, Cambridge, UK.; 12 University of Oxford, Oxford, UK.; 13 Queen Mary University of London, London, UK.; 14 Institute of Biomedicine, Kiinamyllynkatu 10, FI-20014 University of Turku, Finland.; 15 Department of Medical Genetics, Genomics, Laboratory Division, Turku University Hospital, PO Box 52, 20521 Turku, Finland.; 16 Humangenetik Tuebingen, Paul-Ehrlich-Str 23, D-72076 Tuebingen, Germany.; 17 Division of Population Health, Health Services Research and Primary Care, University of Manchester, Manchester, M13 9PL, UK.; 18 Warwick Medical School, University of Warwick, Coventry, UK.; 19 SWOG Statistical Center, Division of Public Health Sciences; 20 Fred Hutchinson Cancer Research Center, Seattle, WA, USA.; 21 Department of Medical Epidemiology and Biostatistics, Karolinska Institute, Stockholm, Sweden.; 22 Department of Applied Health Research, University College London, London, UK.; 23 Centre for Cancer Genetic Epidemiology, Department of Oncology, University of Cambridge, Strangeways Laboratory, Worts Causeway, Cambridge, CB1 8RN, UK.; 24 Division of Cancer Epidemiology and Genetics, National Cancer Institute, NIH, Bethesda, USA.; 25 Division of Nutritional Epidemiology, Institute of Environmental Medicine, Karolinska Institutet, Stockholm, Sweden.; 26 Department of Surgical Sciences, Uppsala University, Uppsala, Sweden.; 27 Division of Public Health Sciences, Fred Hutchinson Cancer Research Center, Seattle, Washington, 98109-1024, USA.; 28 Department of Epidemiology, School of Public Health, University of Washington, Seattle, Washington, USA.; 29 Department of Epidemiology,Harvard T. H. Chan School of Public Health, Boston, MA 02115, USA.; 30 CeRePP and Sorbonne Universite, GRC N°5 AP-HP, Tenon Hospital, Paris, France.; 31 Department of Molecular Medicine, Aarhus University Hospital, Aarhus N, Denmark; 32 Department of Clinical Medicine, Aarhus University & Department of Molecular Medicine (MOMA), Aarhus University Hospital, DK-8200 Aarhus N., Denmark.; 33 Department of Medical Genetics, Oslo University Hospital, Oslo, Norway.; 34 Cancer Epidemiology Unit, Nuffield Department of Population Health, University of Oxford, Oxford, UK.; 35 Center for Genetic Epidemiology, Department of Preventive Medicine, Keck School of Medicine, University of Southern California/Norris Comprehensive Cancer Center, Los Angeles, USA.; 36 Cancer Epidemiology & Intelligence Division, Cancer Council Victoria, Melbourne, Victoria, Australia.; 37 Centre for Epidemiology and Biostatistics, Melbourne School of Population and Global Health, The University of Melbourne, Melbourne, Victoria, Australia.; 38 Fundación Pública Galega de Medicina Xenómica-SERGAS, Instituto de Investigación Sanitaria (IDIS), Santiago de Compostela, Spain.; 39 Biomedical Network on Rare Diseases (CIBERER), Santiago de Compostela, Spain.; 40 Nuffield Department of Surgical Sciences, University of Oxford, Oxford, England.; 41 Department of Oncology, Addenbrooke’s Hospital, University of Cambridge, England.; 42 ISGlobal, Barcelona Institute for Global Health, Barcelona, Spain.; 43 CIBER Epidemiología y Salud Pública (CIBERESP), Madrid, Spain.; 44 IMIM (Hospital del Mar Research Institute), Barcelona, Spain; 45 Department of Experimental and Health Sciences, Universitat Pompeu Fabra (UPF), Barcelona, Spain.; 46 Channing Division of Network Medicine, Department of Medicine, Brigham and Women’s Hospital and Harvard Medical School, Boston, Massachusetts; Department of Epidemiology, Harvard School of Public Health, Boston, MA, USA.; 47 Faculty of Health and Medical Sciences, University of Copenhagen, Copenhagen, Denmark.; 48 Department of Clinical Biochemistry, Herlev and Gentofte Hospital, Copenhagen University Hospital, Herlev, Copenhagen, Denmark.; 49 The Copenhagen General Population Study, Herlev and Gentofte Hospital, Copenhagen University Hospital, Denmark.; 50 Division of Clinical Epidemiology and Aging Research, German Cancer Research Center (DKFZ), Heidelberg, Germany.; 51 Division of Preventive Oncology, German Cancer Research Center (DKFZ) and National Center for Tumor Diseases (NCT), Heidelberg, Germany.; 52 German Cancer Consortium (DKTK), German Cancer Research Center (DKFZ), Heidelberg, Germany.; 53 Division of Medical Oncology, Urogenital Unit, Department of Oncology, University Hospital Centre Zagreb, Zagreb, Croatia.; 54 Molecular Endocrinology Laboratory, Department of Cellular and Molecular Medicine, KU Leuven, Belgium.; 55 Department of Clinical Sciences Malmö, Lund University, Malmö, Sweden.; 56 Department of Public Health and Clinical Medicine, Nutritional Research, Umeå University, Umeå, Sweden.; 57 Department of Biobank Research, Umeå University, Umeå, Sweden.; 58 Department of Medical Biosciences, Pathology, Umeå University, Umeå, Sweden.; 59 Clinical Research Center, Karolinska University Hospital, Huddinge, Sweden.; 60 Icahn Institute for Data Science and Genome Technology, Department of Genetics and Genomic Sciences, Icahn School of Medicine at Mount Sinai, New York, NY, USA.; 61 Department of Chemical Pathology, Pathology Queensland, Princess Alexandra Hospital, Woolloongabba, Brisbane, QLD, Australia.; 62 Department of Anatomical Pathology, Pathology Queensland, Princess Alexandra Hospital, Woolloongabba, Brisbane, QLD, Australia.; 63 Molecular Cancer Epidemiology Laboratory, QIMR Berghofer Medical Research Institute, Herston, Brisbane, QLD, Australia.; 64 Department of Clinical Chemistry, University of Helsinki and Helsinki University Central Hospital, Helsinki, Finland.; 65 Departments of Laboratory Medicine, Surgery (Urology Service) and Medicine (Genitourinary Oncology), Memorial Sloan Kettering Cancer Center, New York, NY, USA.; 66 Department of Translational Medicine, Lund University, Malmö, Sweden.; 67 TissueGnostics GmbH, Vienna, Austria.; 68 Centre for Genomic and Personalised Health, Queensland University of Technology, Brisbane, QLD.

**Keywords:** Prostate cancer, prostate-specific antigen/PSA, diagnosis, Kallikrein-related peptidase 3/*KLK3*, single nucleotide polymorphism, disease aggressiveness

## Abstract

Genetic variation at the 19q13.3 *KLK* locus is linked with prostate cancer susceptibility. The non-synonymous *KLK3* SNP, rs17632542 (c.536T>C; Ile163Thr-substitution in PSA) is associated with reduced prostate cancer risk, however, the functional relevance is unknown. Here, we identify that the SNP variant-induced change in PSA biochemical activity as a previously undescribed function mediating prostate cancer pathogenesis. The ‘Thr’ PSA variant led to small subcutaneous tumours, supporting reduced prostate cancer risk. However, ‘Thr’ PSA also displayed higher metastatic potential with pronounced osteolytic activity in an experimental metastasis *in-vivo* model. Biochemical characterization of this PSA variant demonstrated markedly reduced proteolytic activity that correlated with differences in *in-vivo* tumour burden. The SNP is associated with increased risk for aggressive disease and prostate cancer-specific mortality in three independent cohorts, highlighting its critical function in mediating metastasis. Carriers of this SNP allele had reduced serum total PSA and a higher free/total PSA ratio that could contribute to late biopsy decisions and delay in diagnosis. Our results provide a molecular explanation for the prominent 19q13.3 *KLK* locus, rs17632542 SNP, association with a spectrum of prostate cancer clinical outcomes.

## Introduction

Prostate cancer (PCa) is the second most common malignancy in men world-wide. Serum prostate-specific antigen (PSA) has been the common method of PCa diagnosis for decades^1^. Recent randomized trials^2^ and Screening Trials^3^ showed that PSA testing results in reduced PCa-mortality but also leads to over-diagnosis emphasising the need to revise PSA-based screening for PCa to an individualized, risk stratified and informed decision-making model for men, especially at a younger age. PCa diagnosis by the Free/Total (f/t) PSA ratio, which is lower in PCa compared to those with benign prostatic hyperplasia^4,5^ and other nomograms such as the 4Kscore^6^, are questioned for their clinical utility in discriminating indolent and aggressive PCa and the net benefit these tests add for clinical decision-making^7^.

PSA liquefies semen by cleaving semenogelin proteins^8^ and has a role in tumour progression by cleaving growth factors, and extra cellular matrix (ECM) proteins, increasing migration of PCa cells^9^, angiogenesis^10^ and bone metastasis^11,12^. Genome-wide association studies (GWAS) to date have confirmed that there are now more than 260 single nucleotide polymorphisms (SNPs) that cumulatively explain 42.6% of the familial component of PCa risk in European ancestry^13,14^. Given the clinical importance of PSA in PCa, we and others have earlier performed fine-mapping at the 19q13.3 locus near the *KLK3* [kallikrein related peptidase-3] gene encoding PSA and have shown rs17632542, a non-synonymous SNP (amino acid change Ile to Thr at position 163), is the putative causal SNP at this locus associated with reduced PCa risk^15–18^; however, the exact role of PSA in PCa pathogenesis has not been fully elucidated. Genetic factors may contribute to the differences in serum PSA concentrations and genetic correction to PSA levels may lower the frequency of prostate biopsies^19–21^. Thus, there has been a conundrum as to whether this association of rs17632542 SNP with PCa risk is due to a true biological role of the SNP in PCa pathogenesis or simply reflects the impact of this SNP on PSA measurement, as cases and disease-free controls recruited in most of the GWAS studies have a selection bias based on PSA testing being used to detect the disease.

Herein, we showed that the rs17632542 SNP affects PSA-driven function as seen in *in-vitro* assays and *in-vivo* preclinical xenograft models of tumour growth and metastasis. This suggests there is a plausible biological role for the rs17632542 SNP underlying the risk association finding. Using a suite of biochemical assays, we comprehensively show that the SNP leads to an alteration in the proteolytic activity of PSA, which in turn likely affects the function of PSA in the tumour microenvironment. Our data also indicate that this SNP PSA variant is likely differently detected by the clinically used PSA immunoassays, also affecting the free/total PSA ratio. Furthermore, we explored the association of the rs17632542 variant with PCa risk in three large independent cohorts and identified the SNP to be associated with both PCa risk and survival, but paradoxically, in opposite directions.

## Results

### Thr^163^ PSA has reduced effect on PCa cell proliferation and migration

In terms of risk association for rs17632542, the evidence for how this SNP confers risk is still unclear. We thus explored the impact of PSA variants in controlled *in-vitro* assays. Accordingly, lentivirus vector-based overexpression of furin-recognized PSA isoforms of wild type (Wt) PSA, Thr^163^ variant (encoded by the rs17632542 SNP [C] allele) and Ala^195^ catalytic inactive mutant control (which is an additional control to confirm that the proteolytic activity is important for PSA function); and pCDNA3.1 plasmid vector control (Supplementary Figure 1) was performed for the AR- and PSA-deficient PC-3 cell line. For comprehensive validation, we generated an additional cell line model for lentivirus vector-based overexpression of furin-recognized PSA isoforms (Wt, Thr^163^, Ala^195^ and eGFP) (Supplementary Figure 1) in the AR- and PSA-deficient MSK3 cell line.

**Figure 1.**
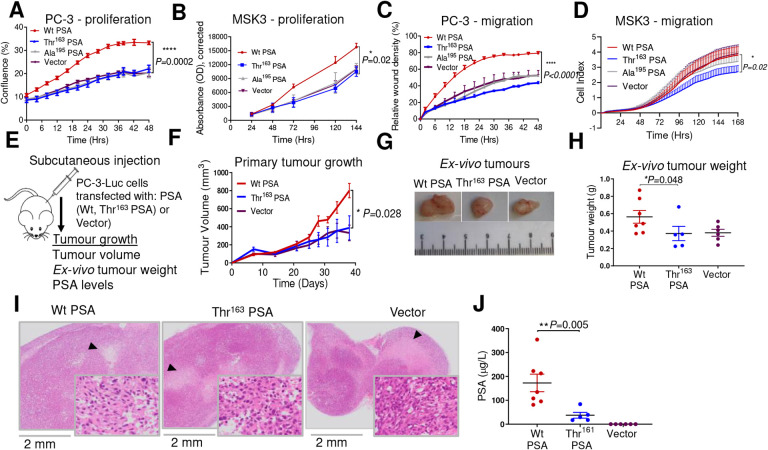
Thr^163^ PSA abolished the effect of PSA on PC-3 cell proliferation and migration and is associated with reduced growth of primary tumours *in-vivo* PC-3 and MSK3 cells were transfected with furin activated Wt PSA, Thr^163^ PSA or control plasmid (vector). **A)** Proliferation rate (confluence %) monitored in the IncuCyte live cell imaging system for PC3 cells expressing PSA variants and vector control over 48 h. Data were consistent across the three independent experiments. ***B)*** Proliferation of MSK3-PSA and vector control cells, measured by Prestoblue cell viability assays. **C)** Cell migration rate (relative wound density %) measured by the IncuCyte live cell imaging system for PC-3 cells expressing PSA variants compared to vector control over 48 h. **D)** Cell migration measured using the xCELLigence system for the PSA variant expressing MSK3 cells as compared to vector control. Three replicates were included unless otherwise indicated from three independent experiments. Data were consistent across the three independent experiments. Data are represented as mean ± SEM. Statistical significance for all these assays were analysed by Friedman test with Dunn’s multiple comparison test. **E**) Preclinical subcutaneous xenograft tumour model of PC-3-Luc cells transfected with furin activated Wt PSA, Thr^163^ PSA or vector (n=7 mice/group). **F)** Mean volume ± SEM of subcutaneous tumours throughout the experiment, based on caliper measurements (mean ± SEM) (Dunnett’s multiple comparisons test). **G**) Representative photographs of resected subcutaneous tumours**. H**) Scatter plot of post-mortem weight of subcutaneous tumours at day 38; horizontal line indicates mean value (mean±SEM) (Mann-Whitney test). **I)** H&E staining of resected subcutaneous tumours. **J)** Serum concentration of total PSA at endpoint (mean±SEM) (Mann-Whitney test).

Expression of Wt PSA in the PC-3 cell line markedly increased the rate of cell proliferation, while that of Thr^163^ PSA did not have any effect (despite their similar expression levels; Supplementary Figure 1), suggesting a high functional impact of the SNP (Figure 1A). As expected, inactive mutant and vector control cells did not show any effect (Figure 1A). Consistently, MSK3 cells transfected with Wt PSA variant exhibited higher proliferation compared to Thr^163^ PSA and vector transfected cells (Figure 1B).

Similarly, while the overexpression of Wt PSA enhanced migration of PC-3 cells, the Thr^163^ PSA overexpression had no effect (Figure 1C). Thr^163^ PSA transfected MSK3 cells also exhibited reduced migration as well as proliferation compared to all three control groups, including Wt PSA expressing cells (Figure 1D). Overall, Thr^163^ PSA transfection exhibited lower cell proliferation, and migration, thus, lacking the activity of Wt PSA.

### Thr^163^ PSA leads to small subcutaneous tumours

Having asserted that the rs17632542 SNP affects the bioactivity of PSA *in-vitro*, we explored the impact of this PSA variant on primary tumour growth in an *in-vivo* context. NSG Mice were implanted subcutaneously with luciferase transfected PC-3 cells expressing Wt PSA, Thr^161^ PSA or eGFP vector control (Figure 1E). PC-3-Wt PSA cells developed the largest tumours (by volume [Figure 1F–G] and weight [Figure 1H]), as observed by day 38, compared to those implanted with PC-3-Thr^163^ PSA cells or vector control PC-3 cells. Necrotic areas were observed in all the tumours (Figure 1I). Serum concentration of total PSA at endpoint was also highest in mice bearing PC-3-Wt PSA tumours (*P*=0.01) (Figure 1J). Collectively, as compared to Thr^163^ PSA expressing cells, Wt PSA expression was associated with higher tumour burden in this preclinical primary tumour model, which correlated with reduced PCa risk for the rs17632542 SNP.

### Thr^163^ PSA increases invasive ability of prostate cancer cells

**Figure 2.**
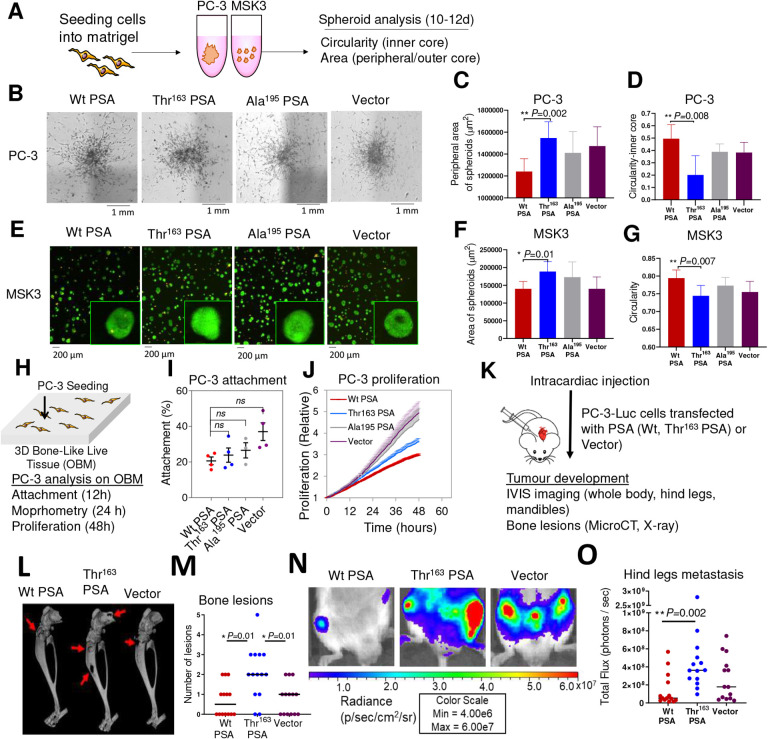
Thr^163^ PSA increased cancer cell invasive ability and increased metastasis *in-vivo* **A)** Schematic workflow of spheroid assay. **B)** Representative brightfield microscope images (4X magnification) of 3D spheroids formed by transfected PC-3 cells after 14 days of culture. **C)** Peripheral area (μm)^2^ of invading cells outside the outer core. Also see Supplementary Figure 2A. **D)** Measure of invasiveness of the spheroid from 0 – 1. A circularity value of 1 (maximum) indicates the spheroid is perfectly circular and least invasive, while a decreasing value towards 0 indicates less circular spheroids. (N = 6 spheroids per condition) **E)** Representative fluorescent microscopy overlay images (10× magnification) of transfected MSK3 cells at 10 days with a magnified view. MSK3 cells were stained with calcein-AM (live cells, green) and ethidium heterodimer (dead cells, orange). Spheroid, Area (μm)^2^ **(F)** and circularity **(G)** were measured. Also see Supplementary Figures 2B, 2C. At least two replicates (2 fields selected per well) from three independent experiments were analysed (Mann Whitney t-test). **H)** Schematic of a 3D osteoblast-derived bone matrix (OBM) co-culture with PC-3 cells, and the various analyses performed. **I)** Attachment of PC-3-mKO2 cells transfected with furin activated Wt PSA, Thr^163^ PSA, Ala^195^ PSA, or vector cells to OBM constructs after 12h co-culture. **J)** PC-3 proliferation on OBM constructs. Also see Supplementary Figures 3A, 3B. For **J**, 2 technical replicates were used, 4–5 fields of view/replicate, for a total of 120–230 cells per condition. *P* values on all groups were evaluated by one-way ANOVA followed by Games-Howell post hoc analysis. **K)** Intracardiac injection of PC-3-Luc-PSA cells in mice (n=7 mice/group). **L**) Reconstructed 3D microCT images of tumour-bearing hind legs from representative mice of each group; red arrows showing areas of significant bone degradation, indicating presence of tumour. **M)** Quantification of bone lesions per hind leg based on visual inspection of planar X-ray images (Dunnett’s multiple comparisons test with Two-way Anova). **N**) Representative bioluminescence images of tumour-bearing hind legs of mice (week 4). **O**) Scatter plots of tumour bioluminescence based on region of interest (ROI) drawn over individual hind legs (at week 4); horizontal line indicates median value (Dunnett’s multiple comparisons test). Also see Supplementary Figure 4.

As three-dimensional (3D) *in-vitro* cell culture systems recapitulate *in-vivo* conditions, we generated anchorage-independent spheroids to analyse the proliferation and invasive potential of the PC-3 and MSK3 cells overexpressing furin-activable PSA variants (Figure 2A). The spheroids’ growth (area of 2D projection and number of spheroids) and invasive ability (circularity/compactness) were analysed (Supplementary Figure 2A, 2B). In Matrigel, preformed PC-3 cell aggregates formed single stellate spheroids, characterized by migration of cells through the surrounding Matrigel matrix (Figure 2B). Thr^163^ PSA expressing PC-3 spheroids showed a higher number of peripheral invading cells (Figure 2C) and less spherical dense inner cores (Figure 2D), indicating a more invasive phenotype compared to Wt PSA expressing spheroids (Figure 2B–D). MSK3 cells, seeded as a single suspension in Matrigel, formed multiple small, circular spheroids (Figure 2E). Thr^163^ PSA expressing MSK3 spheroids showed a higher growth potential with higher spheroid number (Supplementary Figure 2C) and area (Figure 2F), and less circular spheroids (Figure 2G) compared to the Wt PSA expressing MSK3 spheroids (Figure 2E–G). Inactive mutant Ala^195^ PSA and vector transfected cells behaved similarly in respect of all studied parameters in both cell lines (Figure 2B–G). Thus, Thr^163^ PSA expressing cells in spheroid models resulted in a more invasive phenotype suggesting a dual role for the SNP in metastatic dissemination of cancer.

### The Thr^163^ PSA variant differentially modulates PCa cell behaviour in a bone metastasis model

Since PSA has been proposed to promote osteoblastic metastasis^22,23^, a biomimetic *in-vitro* model of PCa metastasis to bone was developed and utilised. Here, stably transfected PC-3-furin activable PSA cells were co-cultured with a 3D osteoblast-derived mineralised matrix (OBM) (Figure 2H). OBM constructs were prepared from patient-derived osteoprogenitor cells and mineralised for 8 weeks, as established previously^24^. Quantitative functional analysis of cancer cell attachment and proliferation on OBM were analysed (Figure 2I–J). After an initial 12h PC-3/OBM suspension co-culture (Figure 2H), PC-3 cells from all groups (Wt PSA, Thr^163^ PSA, Ala^195^ PSA and vector) attached similarly to the OBM constructs (Figure 2I). After a further 12h and 24h co-culture in serum free media, individual PC-3 cells attached to OBM constructs were measured for their shape factor and volume (Supplementary Figure 3A). PC-3 cells displayed significant morphometric differences between groups. Similar to PC-3-vector cells, PC-3-Wt PSA expressing cells did not alter their shape, while a significantly reduced shape factor was observed for the PC-3-Thr^163^ PSA cells (Supplementary Figure 3A). This indicates a more mesenchymal phenotype (spindle-like cell) for PC-3-Thr^163^ PSA cells (*P*=0.02), (as also observed in Supplementary Figure 3B at 24h), which is associated with higher cellular plasticity.

PC-3 cells from all groups colonised the scaffold after 4 days (data not shown) and images taken at 10 days (Supplementary Figure 3B) appeared to demonstrate larger cellular volume for PC-3-Thr^163^ PSA cells on the OBM, when compared to PC-3-Wt PSA cells, possibly owing to a differential substrate specificity for the Thr^163^ PSA. Expression of Wt PSA lowered the proliferation (Figure 2J) of PC-3 cells on OBM constructs compared to Vector cells (*P*=0.03 for proliferation), supporting a tumour suppressive role for Wt PSA in the bone microenvironment. As compared to Wt PSA expressing cells, the PC-3-Thr^163^ PSA cells displayed a more proliferative trend (Figure 2J). Overall, PC-3-Ala^195^ PSA and vector-PC-3 cells behaved similarly throughout all analyses and proliferated more rapidly than both PC-3-Wt PSA and PC-3-Thr^163^ PSA cells (Figure 2I–J, Supplementary Figure 3). Our *in-vitro* data suggests that Thr^163^ PSA expressing cells proliferate at a higher rate in the bone microenvironment in comparison to Wt PSA expressing cells.

### Thr^163^ PSA increased metastasis *in-vivo*

To evaluate the context-dependent effect of the rs17632542 SNP in the tumour microenvironment in bone, here, in an experimental metastasis model, the effects of the furin-activable PSA variants on bone metastasis *in-vivo* were investigated by intracardiac (left ventricular) injection of tumour cells for arterial blood dissemination (Figure 2K). Based on bioluminescence imaging, the liver and kidneys were common sites of soft tissue metastasis, and the hind leg (tibia and femur) and mandible were frequent sites of bone metastasis. The livers (Supplementary Figure 4A), hind legs (Figure 2L–2O, Supplementary Figures 4B & 4C) and mandibles (Supplementary Figure 4D, 4E) of mice injected with PC-3-Thr^163^ PSA cells showed higher number of tumours, which also correlates with whole-body tumour load (Supplementary Figure 4F) and serum PSA levels (Supplementary Figure 4G) compared to those injected with Wt PSA or vector. All three transfected cell lines had the same baseline bioluminescence, as demonstrated by prior *in-vitro* imaging (Supplementary Figure 4H). Collectively, Thr^163^ PSA was associated with highest metastatic tumour burden, including bone metastases, which is consistent with our observation for these cells *in-vitro*, suggestive of a relationship with the poor prognosis of the patients, carrying the rs17632542 SNP, encoding this PSA variant.

### Thr^163^ PSA, has reduced activity towards peptide and protein substrates

Due to this conundrum for both protective and high PCa risk, we wanted to establish if the rs17632542 SNP leading to amino acid substitution Ile to Thr at position 163 of the KLK3/PSA protein sequence, might affect the proteolytic activity of PSA.

**Figure 3.**
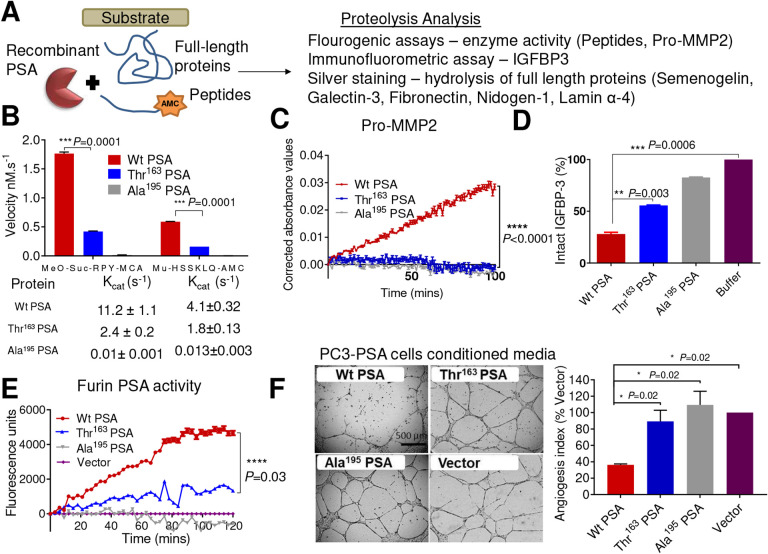
Biochemical characterization of the effect of the Thr^163^ variant on PSA activity. **A**) Schematic for PSA proteolytic activity analysis. **B)** Rate of hydrolysis by mature PSA proteins (Wt PSA, Thr^163^ PSA, and catalytically inactive mutant control Ala^195^ PSA, all at 0.1 μM) were compared using the peptide substrates MeO-Suc-RPY-MCA (10 μM) and Mu-HSSKLQ-AMC (1 μM) over 4 h at 37°C. Proteolytic activity derived from assaying a constant amount of PSA with increasing concentration (0–250 mM) for these two substrates were used to estimate *K*_cat_ values using nonlinear regression analysis in Graphpad Prism. Results are shown as the mean ± SEM from two experiments, each with three replicates (Welch’s t-test ***P=0.0001). Also see Supplementary Figure 5B. **C)** Time (mins) versus relative absorbance (OD) corrected to the substrate alone controls was plotted indicating the activity of pro-MMP2 (0.14 μM) when pre-incubated with PSA protein variants (Wt, Thr^163^ and Ala^195^ at 0.07 μM) at 37°C and then the activity analysed with the chromogenic substrate (Ac-PLG-[2-mercapto-4-methyl-pentanoyl]-LG-OC_2_H_5,_ 40 μM) for active MMP2 over 2 h. Results are shown as the mean ± SEM of three experiments analysed using Kruskal-Wallis test. ****P<0.0001. **D)** Intact/total IGFBP-3 (2.2 μM) after 24h incubation at 37°C with PSA variants (0.25 μM) as shown relative to IGFBP-3 control without added PSA. Also see Supplementary Figure 5C. Results are shown as mean ± SEM (n=3, Welch’s t-test, **P=0.003 and ***P=0.0006). **E)** Fluorescent activity observed for the furin generated active PSA captured from serum free conditioned media of furin-PSA overexpressing PC-3 cells (Wt, Thr^163^, inactive mutant Ala^195^ and vector) using the peptide substrate MeO-Suc-RPY-MCA. (n=2, Mean ± SEM *P=0.03). **F)** Inhibition of HUVEC tube formation on Matrigel by treatment of HUVECs with serum free conditioned media from the PC-3 cells overexpressing (Wt, Thr^163^ and Ala^195^ PSA) and PBS control (negative). Scale bar is 500 μm. The graph to the right represents the effect of these PSA protein variants on HUVEC tube formation expressed as an angiogenesis index. The angiogenesis index, reflecting the extent of tube formation or angiogenic potential of the cells 39,53 is shown in relation to the control (mean + SEM, *P=0.02 as compared to control (t-test), n=2). Also see Supplementary Figure 5D.

Zymography of the recombinant PSA proteins on a casein gel indicated the Thr^163^ PSA variant had lower activity than Wt PSA (Supplementary Figure 5A). Additional proteolytic activity testing (Figure 3A) with two peptide substrates, MeO-Suc-RPY-MCA and Mu-HSSKLQ-AMC (Figure 3B), confirmed that the Thr^163^ PSA had a lower proteolytic activity towards the fluorescent peptides compared to the Wt PSA protein variant and as expected, the mutant Ala^195^ PSA control was inactive. The K_cat_ for Thr^163^ PSA was considerably lower than Wt PSA (Figure 3B and Supplementary Figure 5B).

To further investigate the effect of the Thr^163^ amino acid change on PSA function, we utilised several previously identified substrates of PSA^9,25^. Silver stain analysis after 22 h incubation of recombinant PSA-protein variants with the full-length protein substrates, semenogelin-1, galectin-3, fibronectin, nidogen-1 and laminin α-4 demonstrated that the Thr^163^ PSA had a lower proteolytic activity compared to the Wt PSA (Supplementary Figure 5C). Furthermore, Wt PSA, but not Thr^163^ PSA, can cleave pro-matrix metalloproteinase-2 (MMP2) leading to the activation of zymogen and thus to an active MMP2 protease^26^ (Figure 3C). Similarly, the Thr^163^ variant was less efficient in cleaving the substrate, IGFBP3 compared to Wt PSA (Figure 3D). Together, this data, along with our substrate activity assays, demonstrate that the rs17632642 SNP reduces proteolytic activity of PSA but does not change its substrate specificity.

To confirm whether the PSA secreted (Supplementary Table 1) by the PC-3-PSA cells similarly exhibited a difference in proteolytic activity, PSA was captured by antibodies and activity was measured with the Meo-Suc-RPY-MCA substrate. Again, the measured PSA levels were similar for both the clones although the activity analysis showed the Wt PSA to be more active compared to the Thr^163^ PSA (Figure 3E) similar to our activity analysis with recombinant proteins (Figure 3B).

### The Thr^163^ PSA variant has a reduced anti-angiogenic activity in comparison to Wt PSA

We hypothesised that the pro-metastatic activity of the furin-activable Thr^163^ PSA observed *in-vivo* and altered biochemical activity may reflect the impact of the SNP on the anti-angiogenic role of PSA. Thus, a human umbilicial vein endothelial cells (HUVEC) endothelial tube formation assay was performed using conditioned media from the stable PC-3-PSA cells (overexpressing furin-activable either Wt PSA, Thr^163^ PSA or Ala^195^ PSA) and compared to conditioned media from control cells (PC-3-vector). HUVECs grown on top of Matrigel differentiated into tubular network structures during 16–20 h of incubation. Wt PSA, when incubated with HUVEC cells, showed significant anti-angiogenic activity, decreasing the tube area to 35.2 ± 2.5% (mean ± SD, *P*<0.01) compared to that of the cells treated with conditioned media from control cells (PC-3-vector). The Thr^163^ PSA or inactive mutant Ala^195^ PSA did not show a significant difference in the tube area (88.2 ± 21.0% and 108.4 ± 30.9% of the control, respectively, *P*>0.99 for both) (Figure 3F).

To confirm that the low anti-angiogenic activity, observed against HUVEC cells, in the conditioned media of PC-3-Thr^163^ PSA cells was due to the impact of the secreted PSA, recombinant PSA variants (Wt PSA, Thr^163^ PSA and Ala^195^ PSA) expressed in, and purified from *Pichia pastoris* were utilized in tube formation assays (Supplementary Figure 5D). A similar effect was observed emphasising that the antiangiogenic effect of PSA is dependent on a catalytically functional PSA and that Thr^163^ PSA has a lower antiangiogenic activity compared to Wt PSA (Figure 3F, Supplementary Figure 5D).

### Thr^163^ PSA variant has less complexing ability with serum inhibitors

**Figure 4.**
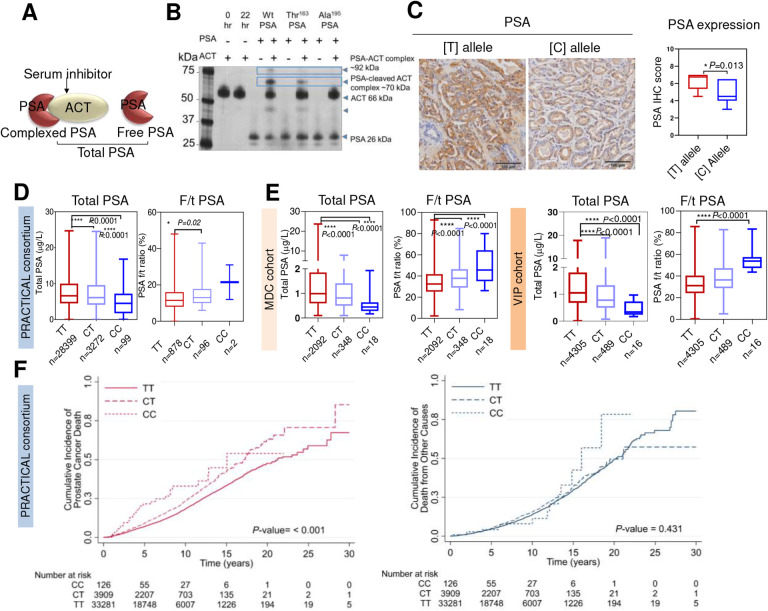
rs17632442 SNP association with PSA levels and prostate cancer survival. **A)** PSA-inhibitor (ACT) complex, free and total PSA. **B)** A representative silver stain analysis of recombinant wild type (Wt) and Thr^163^ and Ala^195^ PSA (0.1 μM) incubated with ACT (0.5 μM) at room temperature for 3 h before resolving on gel showed lower complexing potential of Thr^163^ PSA with ACT compared to the Wt PSA. Inactive mutant Ala^195^ does not complex with ACT. **C)** Representative immunohistochemical results for Gleason Grade 4 adenocarcinoma tissues, showing strong staining for PSA for the TT compared to the CC genotype. Graph on the right shows difference in PSA expression scores between [T] and [C] allele (CC=2, CT=10, TT=10) for the immunohistochemical samples. **D-E)** Genotype correlation of total PSA (tPSA) levels and f/t PSA ratio in prostate cancer cases (PRACTICAL consortium) and disease-free controls (MDC and VIP cohorts). **D)** PRACTICAL consortium. N= 31,770; genotype status TT=28,399, CT=3,272 and CC=99 for tPSA levels comparison. N=976; genotype status TT=878, CT=96 and CC=2 for f/t PSA ratio comparison. **E)** MDC cohort with genotype status TT=2,092, CT=348 and CC=18; and VIP cohort with genotype status TT=4,305, CT=489 and CC=16. (****P<0.0001, Kruskal-Wallis Test). **F)** Survival analysis for the rs17632542 SNP (c.536T>C) in 37,316 cases of PRACTICAL consortium with follow-up on cause specific death. Of these, 4,629 died of prostate cancer, 3,456 died of other causes. Cases by carrier status, TT=33,281, CT=3,909 and CC=126. The cumulative incidence of death from prostate cancer, Hazards ratio (HR)=1.33, 95% CI=1.24–1.45, *P*<0.001 (left panel) and all causes other than prostate cancer, HR=1.08, 95% CI=0.98–1.19, *P*=0.431 (right panel) are indicated. Number at risk are also indicated.

We explored whether rs17632542 affects the complexing ability of PSA with serum inhibitors, thus affecting the f/t PSA that reflects both free PSA, which in blood circulation consists mostly of proteolytically inactive forms, and total immunoreactive PSA, i.e., both free PSA and PSA complexed to its predominant ligand in blood (α-1-antichymotrypsin/ACT/SERPINA3) (Figure 4A). Silver stain analysis of recombinant PSA proteins with recombinant ACT verified a lower complexing ability of recombinant Thr^163^ PSA compared to the Wt PSA as indicated by a lower intensity band of PSA-ACT complex at ~90 kDa compared to the Wt PSA (Figure 4B). An additional band at ~70kDa was observed which could be the PSA complexed with cleaved product of ACT (Figure 4B). Since the complexing ability of PSA with inhibitors may depend on its enzymatic activity, our results are in line with the lower activity observed for the recombinant Thr^163^ PSA protein.

### The rs17632542 SNP [C] allele is associated with low total PSA levels and higher Free/Total PSA ratio compared to [T] allele

Recent studies, including ours, demonstrated that *KLK3/PSA* SNPs were significantly associated with serum PSA levels^18,20,21,27,28^. To confirm the allele specific effect, immunohistochemistry analysis was performed in patient tissue samples (TT=10, CT=10 and CC=2) using an anti-PSA antibody to confirm the allele-dependent expression of PSA at the protein level. Reduced PSA (*P*=0.01) protein levels were observed in tumour formalin fixed and paraffin-embedded (FFPE) slides from patients with the minor [C] allele compared to the [T] allele (Figure 4C).

We analysed the genotype correlation with PSA levels in PCa cases and disease-free controls, since PSA levels may also be influenced by disease grade, stage and age of the individual. We thus assessed the genotype correlation in three independent sample sets. Prostate cancer cases, PRACTICAL consortium (N= 31,770; Figure 4D); disease-free controls, the Malmö Diet and Cancer (MDC) Cohort (n=2,458; Figure 4E) and The Västerbotten Intervention Project (VIP) Cohort (n=4,810; Figure 4E) which indicated lower total PSA (tPSA) levels for the rs17632542 SNP [C] allele.

Among men with modestly elevated PSA, risk assessment based on measuring both f/t PSA and tPSA is considered to have better predictive ability for PCa diagnosis compared to measuring tPSA alone. To explore this further, we assessed the correlation of the rs17632542 [C] allele with f/t PSA ratio available for 958 PCa cases in five cohorts (IMPACT, PRAGGA, PROFILE, TAMPERE and ULM) of the PRACTICAL consortium sample set. In PCa cases, the f/t PSA ratio was 12.82 ± 0.22% for [TT] and 14.67 ± 0.70% for [CT] individuals (mean ± SEM, *P*=0.006) and 21.5 ± 9.5% for individuals with [CC] genotypes (Figure 4D). Similarly, the disease-free men with [CT] and [CC] genotype had significantly higher f/t PSA ratio in both MDC and VIP cohorts (Figure 4E). The f/t PSA ratios were 32.89 ± 0.18 [TT], 38.32 ± 0.64 [CT] and 54.87 ± 2.38 [CC] (mean ± SEM, *P*<0.0001) for VIP cohorts; and 34.11 ± 0.27 [TT], 38.89 ± 0.72 [CT] and 49.57 ± 3.7 (mean ± SEM, *P*<0.0001) for the MDC cohort suggesting that PSA in serum in these men does not form complexes with protease inhibitors as efficiently as with wild type PSA (Figure 4D–4E). Taken together, the [C] allele of the rs17632542 SNP may be associated with poor prognosis for PCa by its synergistic effects on protein expression and clinically measured serum PSA levels.

### *KLK3* rs17632542 SNP is associated with reduced PCa risk but increased metastasis and poor survival

We replicated the association between the rs17632542 SNP and PCa, with an odds-ratio (OR)=0.70, 95% CI 0.67–0.73, (*P=*9.61×10^−69^) for risk of any grade PCa diagnosis in a sample set of 49,941 PCa cases and 32,001 disease-free controls (Supplementary Table 2, Supplementary Table 3) using a custom high-density OncoArray. This association was similar after adjusting for family history (OR=0.75, 95% CI 0.71–0.79, *P*=2.7×10^−26^) and age of disease onset (OR=0.75, 95% CI 0.71–0.79, *P*=5.2×10^−29^) (Supplementary Table 3). The genotype data from this dataset for 46,939 PCa cases and 27,910 disease-free controls of European ancestry was combined with previously genotyped data for 32,255 PCa cases and 33,202 controls (from seven previous PCa GWAS imputed to 1KGP (2014 release)) of European ancestry. Estimated per-allele ORs for meta-analysis of 79,194 PCa cases and 61,112 disease free-controls were similar (OR=0.74, 95% CI 0.72–0.76, *P*= 6.69×10^−81^) and the minor-allele frequency (MAF) of the [C] allele was 0.08. These results suggest that the *KLK3* rs17632542 SNP had a protective effect on PCa risk.

In a secondary analysis for survival within the OncoArray study samples, 37,316 cases were included. Of these 4,629 died of PCa and 3,456 died of other causes (PCa excluded as cause of death). Cases by carrier status were TT= 33,281, CT= 3,909 and CC= 126. Despite the low minor allele numbers, the rs17632542 SNP was significantly associated with PCa specific mortality with a Hazard Ratio (HR) of 1.33, 95% CI=1.24–1.45, *P*<0.001 while for other causes of death HR=1.08, 95% CI=0.98–1.19, *P*=0.4 (Figure 4F). Validation in two independent longitudinal cohort studies of unscreened mid-life men also showed the SNP is associated with high PCa-related death; MDC (HR=1.39, 95% CI=0.98–1.98, *P*=0.06) and VIP (HR=1.69, 95% CI=1.07–2.65, *P*=0.03) (Supplementary Figure 6A, 6B).

Given the rs17632542 is associated with PCa-specific mortality, we analysed if the SNP is associated with aggressive PCa susceptibility. Similar to the cumulative survival analysis, this SNP showed significant differences, but in opposite direction to our initial association analysis; for overall PCa risk, high risk (tumour stage T3/T4 or Gleason Score ≥8 or PSA >20 ng/mL) vs low risk disease (tumour stage ≤T1, Gleason Score ≤6, PSA <10) OR=1.58, 95% CI 1.42–1.76, *P=*1.23×10^−17^, high risk vs intermediate risk (Gleason Score=7, PSA=10–20) OR 1.42, 95% CI 1.33–1.51, *P=*1.41×10^−26^ and risk lethal vs controls OR=1.33, 95% CI 1.16–1.51, *P=*2.29×10^−05^ (Supplementary Table 3). This association predicts whether the SNP is associated with an increased risk of developing advanced stage PCa (tumour stage T3/T4), and therefore a poorer prognosis (Supplementary Table 3). We observed the correlation in a similar direction with risk of PCa death and metastasis-free survival in the VIP cohort. 1,667 prostate cancer cases were selected for this analysis, of which 283 developed metastatic disease during >20 years follow-up. 286 cases were removed during quality control because their observation time was zero. Survival analysis showed rs17632542 is associated with metastasis-free survival time in VIP cohort (HR=1.65, 95% CI=1.03–2.62, P=0.05) (Supplementary Figure 6C). Together, these integrated analyses showed that the [C] allele of rs17632542 SNP is associated with increased risk for aggressive PCa susceptibility and PCa-specific mortality.

For OncoArray study samples where allele distribution with disease status is reported, the distribution of genotype frequency for rs17632542 SNP was calculated. The genotype frequency for this SNP varied with different disease stages as summarized in Supplementary Table 4. We observed higher frequency of the CT genotype at late cancer stage specifically in patients at both N1 (spread of tumour to lymph nodes) or M1 stage (distant metastasis) to be greater (0.15 and 0.13, respectively) compared with early-stage cases (0.10 for both N0 (no spread to lymph nodes) and M0 (no distant metastasis)). Thus, the rs17632542 minor [C] allele is protective against PCa risk overall in a large cohort, which is consistent with previous reports^17,28–30^. However, as we report here for the first time, it is associated with aggressive disease and higher risk of PCa death.

## Discussion

In recent years, the association of SNPs in the PSA encoding *KLK3* gene with PCa risk, PSA levels, or both has been debated, especially since these SNPs appear to influence PSA levels and thus may have influenced patient recruitment in these studies. Thus, characterisation of the biological role may help define their risk association^31^. Here, we present an integrated study explaining the molecular and biochemical function of the protein isoform encoded by the rs17632542 SNP and the clinical implications underlying the *KLK3* PCa risk locus. We identified that the Thr^163^ PSA variant reduces primary tumour growth but is also associated with a higher metastatic tumour burden. This dual risk association for the SNP was supported by our association studies. In men carrying the rs17632542 [C] allele, we observed an overall lower risk of PCa but a higher incidence of PCa-specific death. Notably, the T>C substitution impacts on the expression and proteolytic activity of PSA with synergistic effects on serum f/t PSA levels that could lead to improved prediction of PCa clinical outcome.

The rs17632542 SNP is associated with reduced PCa risk^16–18,32,33^. However, the SNP association with PCa risk or PSA levels remains a conundrum. Thus, characterizing the functional effects may provide more clues to uncover its role in prostate pathogenesis. Thr^163^ PSA expression did not vigorously affect the cellular proliferation and migration of PC-3 cancer cells in controlled *in-vitro* cell-based assays. These results are congruent with those that were obtained from our previous study that described Wt PSA overexpression to increase proliferation and migration of PC-3 cells^34^. Furthermore, the rs17632542 SNP did not affect the growth of primary subcutaneous tumours *in-vivo*, behaving similar to the vector transfected cells, while Wt PSA expression promoted PC-3 tumour growth.

On the other hand, multicellular-spheroids, that mimic tumours *in-vivo* and a 3D bio-engineered osteoblast matrix bone model, allowed us to investigate the effect of the SNP on morphometric properties of PCa cells. Additionally, an *in-vivo* model of metastatic cancer indicated the SNP to lead to the highest metastatic tumour burden, including bone metastasis, compared to Wt PSA. Overall, these analyses support a more invasive capability and phenotype (increased proliferation and more mesenchymal shape) of PCa cells expressing Thr^163^ PSA. Consistently, PC-3 cells expressing Wt PSA showed an opposite trend supporting the notion that Wt PSA may have a tumour suppressive role on these cells in the metastatic tumour context specifically, the bone microenvironment. Our observations are in line with the anti-metastatic role of Wt PSA by hampering adhesion and invasive ability of PCa cells through prostate-derived extracellular matrix^35^. PSA is thought to mediate osteogenesis of mesenchymal stem cells via cadherin-Akt signalling^36^ or affect bone homeostasis through increasing the bioavailability of osteoblastic growth factors such as IGF-1 and modulate genes involved in bone remodelling, such as RUNX-2, osteopontin and TGF-β^22^. PSA may also antagonize the Wnt pathway, by increasing Wnt inhibitory factors and reduce osteoblastic responses to PCa cells^22^. To what extent Thr^163^ PSA can modulate these actions is not yet known but may suggest a differential substrate activity in comparison to Wt PSA.

Treatment of HUVEC cells with Wt PSA reduced their angiogenic potential, but these cellular effects were observed to a lesser degree with Thr^163^ PSA expressing cells or recombinant forms. Wt PSA exerts antiangiogenic activity in endothelial cell models *in-vitro*^37,38^, however, recently it has been suggested to have a lymphangiogenic role as it activates VEGF-C and VEGF-D^10^. This supports previous observations of a dual role of PSA in tumour progression, promoting it by cleaving growth factors and ECM proteins or suppressing it by its anti-angiogenic potential and bone remodelling^39,40^. These studies, however, have only addressed the biochemical capability of PSA, not the bioavailability of PSA and its substrates in the tumour context. Thus, the biological significance of PSA antiangiogenic activity during progression of PCa is not well understood but suffice to say, that in the context of these cell-based models, that Thr^163^ PSA had the reverse effect of Wt PSA.

The proteolytic activity-dependent function of the SNP variant was also apparent in the lower ability to complex with the major PSA binding protein/inhibitor, ACT, a mechanism that requires active PSA^41^. This lower overall substrate binding affinity suggests a possible global structure perturbation that remotely affects the structure of the substrate binding site since the Thr^163^ residue is outside the catalytic site^16^. Notably, SNPs in *KLK3* and other *KLK* genes have been previously related to male infertility^42^. Thus, disruption of PSA proteolytic activity by the Thr^163^-encoding allele may have a substantial impact on the involvement of PSA in PCa pathogenesis. Overall, our findings are consistent with the context-dependent nature of *KLK3* gene function reported by others^43,44^.

A further demonstration of the clinical relevance of rs17632542 was provided by our results in a cohort of PCa patients. The rs17632542 SNP is associated with lower serum PSA levels in our multi-cohort analyses and as reported previously^20,28,29^ supporting a genetic basis for both tissue and circulating PSA levels. Previous studies suggest that the percentage of fPSA contributes to modest diagnostic enhancements above and beyond tPSA alone among men in the “diagnostic gray zone”^45^. High %fPSA was also shown to be associated with worse survival outcome in patients with biochemical recurrence, indicating that fPSA may have role in progression to aggressive disease^46^. Recently, it has been reported that a different biology due to genetic variants underlies the high PCa-specific mortality observed in patients with Gleason Score of 9 to 10 and low PSA levels ≤4 ng/mL^47^. Two SNPs, located in introns 2 and 4 of the *KLK3* gene, and correlated with the rs17632542 SNP (r2>0.8), have been suggested to have potential regulatory effects on *KLK3* gene expression^16^, but their effect on PSA levels has not been addressed to date. Our own recent study has shown that a second non-synonymous rs61752561 SNP in exon 3 of the *KLK3* gene has a potential role in PCa pathogenesis by addition of an extra-glycosylation site, changing protein stability and PSA activity and affects the clinically measured f/t PSA ratio^21^. Our study demonstrates that the rs17632542 SNP is associated with both higher ratios of f/t PSA due to its effect on reducing the ability to complex with inhibitors (PSA-ACT complexes), as well as lower levels of tPSA in blood which is expected due to the higher ratio of f/t PSA and much shorter clearance rate from blood compared to complexed PSA^48^. The lower PSA levels among the C-allele rs17632542 variant men are more likely prone to: 1) a negative detection bias as fewer of these men would be referred to prostate biopsies and; 2) due to this PCa-detection bias, more likely to be diagnosed with more advanced disease stages as their referral for a biopsy would be delayed due to a more modest PSA elevation and a higher ratio of free-to-total PSA. The high f/t PSA ratio may explain the protective effect of the C-allele rs17632542 variant in reference to risk of any grade prostate cancer diagnosis.

The [C] allele of the rs17632542 SNP has been documented to be associated with lower PSA levels^16,28^, reduced tumour volume^33^ and reduced PCa risk^16,18,30,49^. This correlated with the risk association overall for the SNP in a large multicentre patient analysis herein, of which the major proportion of men contributing have low-grade disease. Survival analysis revealed poorer prognosis for the patients carrying the [C] allele in our multiple cohort-PRACTICAL study and two additional independent MDC and VIP cohorts. We therefore identified, for the first time, the rs17632542 SNP [C] allele to be associated with PCa-specific death. We compared, high risk or fatal PCa and low risk disease and metastasis-incidence and found the [C] allele is associated with an increased risk of developing metastatic disease with the SNP allele more frequent in patients who have tumour spread to lymph nodes (N1) or distant metastasis (M1).

Our study adds substantially to previous studies by indicating the potential for considering integration of SNPs with PSA into diagnostic pathways. By applying genetic correction of PSA levels using 4 SNPs including the rs17632542 SNP, 6–7% of Icelandic men undergoing PSA screening, would have at least one PSA measurement reclassified with respect to whether they have to undergo prostate biopsy^28^. Using the same four PSA-SNPs it was suggested that, nearly 18–22% of unnecessary biopsies may be reduced by genetic correction^19^. While there is substantial evidence demonstrating that the genetic background of individuals rather than SNPs within PSA can influence PSA levels, for the first time, our study provides functional effects of germline variants on PCa tumorigenesis. Since the rs17632542 SNP is associated with poor survival, it is critical to carefully monitor men carrying either of the CT or CC genotypes as they may have aggressive cancer, without having abnormal total or f/t PSA values.

The current study has several important strengths. The identification of the rs17632542 SNP was based on a validation in several large-scale independent studies. To date, the relationship between PSA SNPs and PCa risk has remained obscure. We carefully applied gene overexpression strategies and clarified the functional and phenotypic relevance of the rs17632542 SNP with PCa pathogenesis making the association between the germline variant and PCa susceptibility biologically plausible. The rs17632542 SNP, although associated with reduced PCa risk, is also associated with an aggressive phenotype and PCa-mortality. The rs17632542 SNP contributes to reduced expression of transcript and serum PSA levels that may lead to detection bias during PSA screening leading to delayed diagnosis and treatment. Thus, these findings, may allow better prognostic prediction, and in distinguishing a more lethal phenotype, to identify a high-risk group that need early treatment regimens. Combination of this SNP effect with other genetic variants reported recently^50,51^ would also facilitate more accurate prediction of PCa risk. In our study we have observed the Wt PSA to have a protective role during PCa metastatic progression although the biology underlying the higher metastatic potential for the Thr^163^ PSA still needs further investigation.

## Online Materials and Methods

### Mammalian cell culture

The androgen-independent bone metastasis-derived human PCa cell line, PC-3 and HUVECs (for studying PSA variants secreted into conditioned media by PC-3-PSA cells), were purchased from the American Type Culture Collection. HUVEC cells, isolated from umbilical veins, were cultured as described previously^52,53^. PC-3 cells were maintained in RPMI-1640 medium supplemented with 5% FBS and passaged using Versene (Invitro Technologies) in an atmosphere of 5% CO_2_ and 99% relative humidity at 37°C. MSK3 cells, a mucinous adenocarcinoma isolated from a retroperitoneal lymph node generated at Memorial Sloan-Kettering Cancer Center^54^ and resourced through Dr Ian Vela, Queensland University of Technology, were maintained in a serum-free conditioned prostate culture medium as previously described^55^ and passaged using TrypLE (Invitro Technologies). All cell lines were tested for mycoplasma. With respect to their genotype status for the rs17632542 SNP, PC-3 is heterozygous CT genotype, while MSK3 is homozygous TT genotype (data obtained through RNA sequencing data of the native cell lines). Androgen-dependent PCa cell lines such as LNCaP, DUCaP and VCaP secrete high PSA levels and so could not be used for this analysis.

Human mesenchymal osteoprogenitor cells were isolated from bone tissue obtained under informed consent from male patients undergoing hip or knee replacement surgery (QUT ethics approval number 1400001024), as described previously^56^. Cells were cultured in growth media (GM), containing alpha-Modified Eagle Medium (alpha-MEM), supplemented with 10% Fetal Bovine Serum (FBS), 100 U/mL penicillin and 100 μg/mL streptomycin (all from ThermoFisher Scientific). Cells were used at passages 3–5 and were mycoplasma free.

### Construction of plasmids for PSA variant expression

To ensure the activation of the expressed PSA, the expression constructs were engineered by changing the region encoding the pro-domain (APLILSR) of the PSA sequence to one encoding a furin recognition sequence (APLRLRR)^57^. The pcDNA3.1-PSA vectors encoding furin activatable Wt PSA were generated as previously described^21^. Site-directed mutagenesis was performed to create the SNP allele isoform, Thr^163^ PSA, and catalytically inactive (Ala^195^ PSA) PSA isoform using mutagenic primers (Supplementary Table 5). Mutated PSA sequences were confirmed by Sanger sequencing using T7 and BGH primers (Supplementary Table 5). To generate lentiviral vectors, pCDNA3.1-PSA (Wt and Thr^163^) vectors generated above were used as template and amplified using attB overhangs and subsequently cloned into a pLEX307-Puro overexpression plasmid. The pLEX307-GFP plasmid used as a control was kindly provided by Dr Sally Stephenson (Queensland University of Technology, Australia).

To generate luciferase-labelled PSA-expressing PCa cells for *in-vivo* models, PC-3 cells were transfected via a lentiviral vector-base method. cDNA encoding luciferase protein from a pGL4.10-luc2plasmid (Promega, Sydney, Australia) was cloned into a pLenti CMV Hygro DEST vector (Addgene, Cambridge, MA) using Gateway LR recombination cloning technology (Life Technologies). Cells stably infected with the luciferase construct were selected in hygromycin (1 mg/ml) containing medium.

### Cell models for expressing Wt and PSA variants

PSA constructs generated as described above were transfected into PC-3 and MSK3 cells (50,000 cells) seeded into 24 well plates using the FuGENE^®^ transfection reagent (Promega) according to the manufacturer’s instructions (1:3 ratio of DNA to lipid used). PSA expression levels by the PC-3/MSK3-PSA polyclonal populations were tested by qRT-PCR (PSA primers: Supplementary Table 5) and Western blot analysis using an anti-PSA antibody (Dako, A0562) before subsequent characterisation below. For evaluating the morphological effect of the PSA variants on bone scaffolds, the PC-3-PSA cells were re-transfected with the pLEX307-mKO2 plasmid (a kind gift by Dr Sally Stephenson), sorted by Fluorescence Assisted Cell Sorting (FACS), and verified for PSA expression prior to use.

For lentiviral viral transduction, lentiviral particles were generated in HEK293T host cells transfected with FuGENE^®^ transfection reagent (Promega) and pLEX307-fPSA/Vec plasmids generated above. The pCMV-8.2R lentiviral packaging plasmids and pCMV-VSVG were kindly provided by Dr Brett Hollier (Queensland University of Technology, Australia). Virus particles were collected after 48 h of transfection and filtered through a 45 μm filter.

### qRT-PCR for PSA expression analysis

Total RNA was extracted from PC-3 and MSK3 PSA overexpressing and vector cells using the Isolate II RNA mini kit (Bioline, Australia) according to the manufacturer’s instructions. One μg of RNA was reverse transcribed using SuperScript III reverse transcriptase (Invitrogen) and amplified using the SYBR Green PCR Master Mix (Applied Biosystems^®^). The primer sequences are listed in Supplementary Table 5. Relative expression levels of the target genes were determined by the comparative C_T_ (ΔΔC_T_) method^58^.

### DELFIA^®^ immunofluorometric assay for free and total PSA

The secretion of PSA in the serum free RPMI conditioned media of PSA transfected clones (PC-3-PSA) was determined with a dual-label DELFIA immunofluormetric assay (PROSTATUS^™^ PSA Free/Total PSA from Perkin Elmer, Australia). Briefly, the PSA in the conditioned media was captured to the immobilised anti-PSA antibody and the free to total PSA ratio was calculated^59,60^.

### *In-vivo* mice models

#### Animal Ethics statement

All studies were performed in accordance with guidelines of the Animal Ethics Committees of The University of Queensland (AEC number: 091/17) and Queensland University of Technology, and the Australian Code for the Care and Use of Animals for Scientific Purposes. Male NOD SCID gamma (NSG) mice, 5–6 weeks old (n=7 mice/group), were sourced from the Australian Resources Centre (ARC; Australia). All mice were maintained at the Biological Resources Facility at the Translational Research Institute, Woolloongabba, QLD.

#### In-vivo tumorigenesis studies

Subcutaneous implantation of 1×10^6^ PC-3-Luc-furin activatable PSA (Wt, Thr163)/Vec cells in PBS was performed on the right flank of mice in 100 μL volume. The tumours were measured using electronic calipers every 2–3 days and tumour volume calculated from the formula for the volume of an elipse: V  =  π/6(d_1_.d_2_)^3/2^, where d_1_ and d_2_ are two perpendicular tumour dimensions. In the metastasis model, cells were injected into the left ventricle of mice for arterial blood dissemination, a technical procedure guided by a small animal ultrasound imaging station (Vevo 2100, Visualsonics, Canada) as described^61^

#### Tumour bioluminescence imaging

Tumour development was monitored by weekly bioluminescence imaging using an IVIS Spectrum (Perkin Elmer, USA). For *in-vivo* imaging, mice were injected intraperitoneally with D-luciferin diluted in PBS (15 mg/ml stock) at 150 mg/kg, anaesthetised and imaged until tumour bioluminescence plateaued. Bioluminescence was analysed using Living Imagine software (Xenogen, CA, USA). The total flux in photons/second (p/s) within each defined region of interest (ROI) provides a surrogate of tumour burden. For *in-vitro* imaging, bioluminescent cells were seeded at 50,000 cells/well down to 50 cells/well (2-fold serial dilution) in 96-well plates. D-luciferin (Perkin Elmer, USA) was added to each well (final concentration was 150 μg/mL of media) 3–5 mins prior to imaging.

#### High resolution microCT (ex-vivo)

High resolution microCT imaging was performed using a Skyscan 1272 (version, 1.1.19; Bruker, Belgium). Mouse leg specimens were fixed in 10% neutral-buffered formalin for 48 hours, stored in 70% ethanol, then wrapped in moist tissue paper and transferred into 5 mL cylindrical plastic tubes for imaging. The scanning parameters were: 70kV X-ray voltage, 142 uA current, 600 ms exposure time, 19.8 μm isotropic voxel size, 0.5° rotation step (360° imaging), 2 frame averaging, 4×4 binning, and 0.5 mm Al filter. The datasets were reconstructed with NRecon (Bruker) and InstaRecon (University of Illinois, USA) software using cone beam reconstruction (Feldkamp) algorithm and the following corrections applied: ring artefact reduction, beam hardening, and post-alignment. CT analysis was performed using CTAn software version (Bruker), and 3D visualisations of legs generated using CTVox software (Bruker).

#### X-ray Radiography (ex-vivo)

Post-mortem X-ray imaging of resected mouse hind leg bones was performed using a Faxitron Ultrafocus digital X-ray system (Faxitron Bioptics, USA).

#### Histologic analysis of mouse tissues

Subcutaneous tumours and tumour bearing tissues for metastasis models harvested *ex-vivo* were fixed in 4% paraformaldehyde. Histologic analysis was performed for confirming the presence of tumour cells in specific organs and mice hind legs at the end of the experiment. Bone specimens were decalcified in 10% EDTA in PBS for two weeks and the decalcified bones were separated and embedded in paraffin blocks. Serial sections of both subcutaneous tumours and mice legs with metastatic lesions were stained with hematoxylin and eosin (H&E). Tartrate-resistant alkaline phosphatase staining was performed using an Acid Phoshphatase kit (Sigma Aldrich) according to the manufacturer’s instructions.

### Analysis of cell proliferation, migration and invasion of PSA variant expressing cells

For PC-3 cell proliferation analysis, 5,000 PSA variant transfected cells were seeded overnight in 96-well flat-bottomed plates and monitored in the IncuCyte live cell imaging system (Essen Biosciences) in serum free conditions over 48–72 h. Proliferation for PSA variant transfected MSK3 cells was assessed using PrestoBlue reagent (Invitrogen, Australia) and CyQUANT cell proliferation assay (ThermoFisher Scientific, Australia). For the PC-3 cell migration assay, 3×10^4^ cells were plated per well in a 96-well ImageLock plates (Essen Biosciences) and incubated overnight at 37°C (Sigma Aldrich) to form a confluent monolayer of cells. The cells were pre-treated with Mitomycin-C (at 10 μg/mL) for 2 h before a scratch was made using a 96-pin WoundMaker^™^ (Essen Biosciences). For PC-3 cell invasion, 96-well ImageLock plates were pre-coated with 100 μg/mL of phenol-red free growth-factor reduced Matrigel^®^ matrix (Corning, USA), 3×10^4^ cells allowed to adhere and 50 μL of Matrigel Matrix (1 mg/mL) added on top of the cell monolayer prior to wound making. Migration and invasion were measured in either serum free media or media with 5% FBS over a period of 48–72 h using the “Relative Wound Density” metric generated by IncuCyte software. Migration for MSK3 cells were assessed using the xCELLigence system according to the manufacturer’s instructions (Roche) by plating 5×10^4^ cells per well and cell index/time was derived using the RTCA software. At least three technical replicates per group were included. In total three biological replicates were performed.

### 3D spheroid cell models and morphological analyses

To monitor changes in invasiveness and tumour-specific differentiation patterns, MSK3 cells transfected with furin-activatable PSA variants were embedded between two matrigel layers (4,000 cells/well). After 10 days in 3D culture, live/dead staining was performed using Calcein AM live cell dye and ethidium homodimer (both from ThermoFisher Scientific, Australia), respectively. Stacks of images of MSK3 spheroids were taken with an INCell Analyzer 6500 HS high content analysis system (GE Healthcare Life Sciences, Australia). PC3 spheroids were generated in a Matrigel matrix. Briefly, PSA variant transfected PC-3 cells were plated at 1000 cells/well on an Ultra-low Cluster 96 well plate (Sigma Aldrich, Australia) in low FBS (2%) containing RPMI media. After 4 days, 100 μL of phenol-red free growth-factor reduced Matrigel^®^ matrix (Corning, USA) (10 mg/mL) was added to each well, topped up with 100 μL media (2% FBS) after 1 hour and incubated for 10 days. A Z-stack of 30–50 focal images, with a step-size of 25 μm between layers was acquired using a Nikon spinning disc confocal microscope and 4× objective. Digital analysis was acquired for the images on spheroid - number, size, morphology (circularity/compactness) and viability of spheroids (live - Calcein/green and dead -heterodimer/orange staining) were quantified using a custom analysis pipeline in the StrataQuest^™^ image cytometry software (TissueGnostics, Vienna, Austria) to automate the quantitative analyses of spheroids.

For PC-3 spheroids, live/dead cell staining could not be performed since we observed a high background of calcein staining the matrigel. To perform image cytometry several analysis engines were defined in the image analysis environment, StrataQuest, to process the original Tiff images in a pipeline process. A grey channel image was generated from the original image. The background was removed from the grey image to correct for illumination artefacts using a set of engines to locally reduce the background. Then a threshold was applied to the images for the detection of positive objects and a density image was generated. The high-density area was split into two parts – the inner core and outer core based on intensity. The periphery is set as the complement of the two parts. Three areas were generated ash shown in Supplementary Figure 2A, with the green contour overlay highlighting the outer core and the orange contour overlay indicating the area of the inner core. The blue contour overlay around the spheroids contains detectable cells in the periphery. Finally, after the recorded images of single PC-3 stellate spheroids per well were segmented, several measurements were performed. The read-out parameters includes the circularity of the dense central/inner core of each spheroid, the area of the outer core and the area of the peripheral invading cells, to indicate invasive ability. Manual correction was performed to remove artefacts, where necessary, to assure data consistency.

For MSK3 spheroids that formed multiple circular spheroids, circularity of the whole spheroids and additional properties such as live/dead cell staining, number of spheroids and maximum intensity projections created from z-stacks were determined. The original Tiff images, in sets of two 16-bit grey scale images, one each for the green Calcein and the orange ethidium homo-dimer markers were used. Dead cells were detected using a combination of two detection engines. First a detection of dot like structures, with high intensity in the centre and lower (gradient) intensity around the centre. The second step was a detection of specific stained areas / marker positive cells using an intensity thresholdoperation. Both segmentation masks were merged for a final detection of the dead cells. Spheroids were detected based on a double threshold on intensity and area. (Supplementary Figure 2B).

Statistics were generated automatically based on total event count as measure of spheroid number, count, and mean intensity for live and dead cells within the spheroid and event area for spheroid area. Manual correction for automatic cell detection was performed for single live/dead cells, where necessary, to compensate for air bubbles and other erratic background patterns. At least two technical replicates per group were included. In total three biological replicates were performed.

### Co-culture models of osteoblasts with PC-3- PSA -mKO2 expressing cells

#### Scaffold Fabrication

Microfibre scaffolds made of medical-grade polycaprolactone (mPCL) were produced by melt-electrospinning with an in house-built equipment and protocol^24^. Final scaffolds were 12×12×0.4 mm in size, with a 3D interconnected structure and 150 μm pore size. Scaffolds were coated with calcium phosphate to promote cell adhesion and osteogenic differentiation, as described previously^62^.

#### Scaffold Culture

After sterilization with 100% Ethanol and UV radiation (20 min both sides), mPCL scaffolds were seeded with osteoprogenitor cells (800,000 cells/scaffold) in a 5 μL drop in the centre of the scaffolds. After attachment (4 h), scaffolds were cultured in growth media (GM) (MEM-Alpha with 10% FBS and 1% Penicillin/Streptomycin) until they reached 3D confluency within the scaffold. Media was then changed to osteogenic media (OM), containing GM + 10 nM β-glycerophosphate, 0.17 nM ascorbic acid, 100 nM dexamethasone (all supplied from Sigma-Aldrich, Australia) and scaffolds were cultured for 8 weeks until mineralization occurred. Media change was performed 2 times a week with fresh OM made weekly. The final osteoblasts/scaffold constructs are referred to as ‘OBM constructs’ and displayed relevant bone characteristics (collagen deposit, mineralization) as demonstrated previously^62^.

#### OBM Co-Culture with PC-3-PSA-mKO2 Cells

Once mineralised, OBM constructs were washed in serum-free RPMI media 3 times. Biopsy punches (5 mm) were made from the constructs and placed in a 24 well-plate prior to seeding of PC-3 cells overexpressing Wt, Thr^163^, Ala^195^ PSA or Vector, re-transfected with pLEX307-mKO2. PC-3-PSA-mKO2 cell solutions were prepared in serum-free RPMI at a concentration of 50,000 cells/mL. 500 μL was seeded on the scaffolds (25,000 cells total/well) and incubated (37°C, 5% CO2) overnight on a shaking platform. Upon PC-3-mKO2 cell attachment to OBM constructs (12 h), cell suspensions were removed and counted to determine the degree of PC-3 attachment to OBM, and the constructs were washed 3 times with serum-free RPMI. PC-3/OBM co-culture (CC) constructs for morphometry were then placed in new 24 well-plates and cultured for a further 12h in serum-free RPMI. CC constructs were then washed 3 times in serum-free RPMI and fixed in 4% paraformaldehyde for 3h, followed by 3 washes in PBS and stored at 4°C until staining. Quantitative functional analysis of cancer cell attachment, morphometry, and proliferation on OBM has been established previously for PCa cell lines^63^, and was applied here.

For proliferation, some CC constructs were used for live cell imaging for a further 48 h in serum-free conditions, after the initial 12h attachment. For long-term cultures, CC constructs used for live imaging experiments were further cultured in 5% FBS-RPMI up to 10 days. While in culture, CC constructs were monitored with an Olympus BX60 microscope using a CY3 (red) filter to identify PC-3-mKO2 cells on OBM, and bright field for general topography. After 10 days, CC constructs were washed 3 times in serum-free RPMI and fixed in 4% paraformaldehyde for 3 h, followed by 3 washes in PBS and stored at 4°C until staining.

#### Immunofluorescence Staining

PC-3/OBM constructs were stained by DAPI (5 mg/mL) for nuclei staining and Alexa Fluor Phalloidin 488 for actin staining (0.8 U/mL), (ThermoFisher Scientific, Australia), diluted in 0.5% Bovine Serum Albumin (BSA) in PBS (Sigma-Aldrich, Australia). Constructs were incubated for 45 min at room temperature with the staining solution, rinsed 3 times in PBS (10 min per rinse) on a shaking platform. Constructs were transferred to 2 mL Eppendorf tubes supplemented with fresh PBS and stored at 4°C until analysis.

#### Fixed Imaging

PC-3/OBM constructs were imaged for morphometry (fixed after 1 day co-culture) and for overall morphology (fixed after 10 days co-culture), on a Nikon Spectral Spinning Disc Confocal microscope (X-1 Yokogawa spinning disc with Borealis modification) fitted with a 10X PlanApo objective, using green (FITC, excitation at 488 nm, laser power at 72%, exposure time 300 ms, Gain 1.5×), red (CY3, excitation at 561 nm, laser power at 73%, exposure time 400 ms, Gain 1.5×) and blue (DAPI, excitation at 405 nm, laser power at 54%, exposure time 100 ms, Gain 1.5×) filter sets. Z-stacks were obtained from 51 images taken every 1 μm over a 50 μm thickness, comprising the PCa cell layer on top of the OBM. Four different fields of view were collected for morphometry analysis per CC construct and 2 constructs/condition were used.

#### Live Cell Imaging

Live PC-3/OBM constructs were placed in a 24 well plate in serum-free conditions (500 μL) and secured down used Teflon rings. An Olympus Live Cell microscope was used to record videos of cells for 48h. Images were taken every 20 min (4X objective) using CY3 (red) to identify PC-3-mKO2 cells moving on OBM, and bright field channels for general topography. Videos were reconstructed from images (145 frames in total). An average of 8 fields of view were recorded per CC construct and 2 constructs/condition were used.

#### Image Analysis

For morphometric and migration studies, images were analysed using Imaris imaging analysis software (Version 8.4.1, Bitplane AG, Zurich, Switzerland). For morphometric analysis (cellular volume and sphericity), automated surface statistics were computed from Z-stacks (algorithm parameters: Surface area detail 1 μm, Threshold: Automatic, Diameter 11 μm, Quality filter: Automatic) for at least 100 cells per group. For migration analysis (speed), automated spots statistics were computed from live cell imaging series (algorithm parameters: Estimated cell diameter 18 μm, Intensity filter 30–230, Max distance jumps 20 μm, Max gap size 5) for 120–230 cells/tracks per group. For proliferation studies, live cell image series were analysed using ImageJ software (Version 1.51h, Rasband, W.S., ImageJ, U. S. National Institutes of Health, Bethesda, Maryland, USA). In brief, the area occupied by PC-3 cells at each time point was measured by setting a high intensity threshold for the mKO2 (red) signal and using the area measurement function of ImageJ. An average of 8 fields of view were recorded per CC construct and 2 constructs/condition were used.

### Production of recombinant active PSA

For recombinant protein overexpression in *Pichia pastoris*, *KLK3* cDNA (NCBI RefSeq: NM_001648.2) previously cloned in the pCDNA3.1/V5–6His vector^34^ was engineered to include a pre-signal sequence for secretion in *Pichia pastoris* and then cloned into the pPIC9K vector (Invitrogen) conferring a N-terminal enterokinase and hexahistidine (6His) tag. Single point mutations were generated using mutagenic primers to generate the Ile163Thr (Thr^163^ PSA) and Ser195Ala (Ala^195^ PSA) substitutions followed by expression in *Pichia pastoris* GS115 cells as described previously^64^.

Transformants expressing high levels of each of the protein variants were chosen for larger scale expression and purification by cation exchange chromatography and the purified proteins were further subjected to enterokinase (EK) digestion and purified by cation exchange chromatography as described previously^65^.

### *In-vitro* enzymatic assay for the secreted PSA and variants

Secreted PSA in conditioned media was captured on a 96 well plate by a PSA specific antibody (PROSTATUS^™^ PSA Free/Total PSA from Perkin Elmer, Australia) immunoassay as described above. The activity of the captured PSA specific was determined by the addition of a fluorescent peptide substrate (MeO-Suc-RPY-MCA, 1 μM/well/200 μL) diluted in TBST assay buffer (0.1 M Tris base pH 7.8, 0.15 M NaCl, 10 mM CaCl_2_, 0.005% Triton X-100). The plate was incubated with slow shaking at 37°C and fluorescence was measured at 355 nM (excitation) and 460 nm (emission) every 3 mins for approximately 4 h. Three technical replicates per group were included. In total three biological replicates were performed.

### PSA activity assays with peptide and protein substrates

#### Determination of PSA enzyme activity

The enzymatic activity of the recombinant PSA variants was measured using two fluorescent peptides (MeO-Suc-RPY-MCA^66^ (Peptides International) and Mu-HSSKLQ-AMC^67^ (Sigma Aldrich, Australia). Fluorogenic assays were performed in 384-well microplates (Corning). PSA proteins (0.1 μM) were incubated with 1–10 μM fluorogenic substrates in 50 mM TBST buffer for the MeO-Suc-RPY-MCA substrate or TBS (0.1 M Tris base pH 7.8, 0.15 M NaCl, 10 mM CaCl_2_) with 0.1% BSA for the Mu-HSSKLQ-AMC substrate. The plates were incubated for 4 h at 37°C and fluorescence was measured at 355/460 nm (excitation/emission) with a POLARstar Omega Plate Reader Spectrophotometer (BMG labtech). Three technical replicates per group were included in three independent biological replicates.

The V_max_ (maximum rate of reaction), *K*_m_ (Michaelis constant) and *K*_cat_ (catalytic rate constant) were determined for PSA with both peptide substrates (0–250 mM) using non-linear regression analysis in the GraphPad Prism software. Velocity (V) was calculated from the change in fluorescence/min at the linear phase of the reaction and the Relative Fluorescence Units (RFU) was transformed to molar concentrations by a standard curve for 7-amido-4-methylcoumarin (AMC, Sigma Aldrich).

#### PSA activity on protein substrates

Recombinant protein substrates semenogelin, fibronectin, nidogen-1, laminin α-4 and galectin-3 (R&D Systems) (0.5 μM) were incubated with mature 0.2 μM recombinant PSA (Wt, Thr^163^ and Ala^195^) at 37°C for 18 h in TBST buffer and analysed by SDS-PAGE as previously described^21^. The activation rate of pro-MMP2 (0.14 μM) and hydrolysis of IGFBP-3 by the PSA protein variants (0.07 μM) was determined by a MMP2 screening assay (Abcam) and an immunofluorometric assay to detect intact and total IGFBP-3, respectively, as described previously^21^. Three technical replicates per group were included. In total three biological replicates were performed.

### HUVEC angiogenesis assays to analyse the anti-angiogenic potential of PSA

The antiangiogenic activity of the PSA protein variants was assessed by the HUVEC tube formation assay as described previously^9^. HUVECs were used for tube formation experiments until passage 8^53,68^. Briefly, four-chamber cell culture slides were coated with Matrigel^™^ basement membrane preparation (BD Biosciences) and HUVECs (1.2×10^5^) were added on top of the Matrigel and incubated with conditioned media (200 μL/well) from the stable transfected PC-3-PSA cell line models which were serum starved prior to performing the angiogenesis assay. HUVECs were grown on Matrigel for 18 h, before live cell images were taken using the EVOS fluorescent microscope (AMG, Mill Creek, USA). Five (2× objective) to 14 (4× objective) live cell images for each cell culture chamber were analyzed by Fiji ImageJ 1.50b^69^ using Angiogenesis Analyzer macro^70^. The following measurements were included in the analysis of angiogenesis index: number of junctions, master junctions, master segments, sum of the length of the detected master segments, and number of meshes and sum of mesh areas detected in the analyzed area. Angiogenesis index, reflecting the extent of tube formation or angiogenic potential of the cells, was defined as the average of all these parameters (in relation to control). The angiogenesis index was in keeping with the visual inspection of the images and also with the previously described effect of PSA-B in HUVEC tube formation^9,52^. Similarly, the anti-angiogenic potential observed with conditioned media from PSA overexpressing PC-3 cells was verified by 250 nM of the recombinant PSA protein variants Wt, Thr^163^ and Ala^195^. Control wells contained an equal amount of phosphate buffered saline (PBS) in culture medium. At least two technical replicates per group in two biological replicates were performed.

### Analysis of the PSA-ACT complex

To analyse the effect of the rs17632542 *KLK3* SNP in complexing of PSA variants with ACT (predominant PSA inhibitor in serum), 0.1 μM recombinant mature PSA proteins (generated in *Pichia pastoris*), Wt, Thr^163^ and Ala^195^ were incubated with ACT (0.5 μM) for 15 mins at RT, denatured at 70°C for 10 min and samples were analysed by SDS-PAGE followed by silver staining.

### Immunohistochemical analysis of patient tissues

FFPE blocks from prostate tumours (n=23) were obtained from the Australian Prostate Cancer Bio-Resource tumour bank. These patients were genotyped for the rs17632542 SNP in our Illumina iSelect genotyping array (iCOGS). A detailed summary on the genotype, age at diagnosis, family history, Gleason Grades, Gleason Score and PSA levels at diagnosis were obtained. Immunohistochemical (IHC) staining was performed using FFPE sections (4 μm) incubated with anti-PSA antibody (1:5000) (Dako) overnight at 4°C followed by incubation with anti-rabbit goat DAB-polymer-linked secondary antibody-based detection (Dako) according to the manufacturer’s instructions. Images were acquired using an Olympus VS120 Brightfield slide scanner. All IHC samples were assessed by two independent researchers (a pathologist and an IHC expert) blinded to subject outcomes and sample origin. Each slide was scored for the percentage of PSA positive cells (0% positive cells=0; 1–25% positive cells for 1; 26–50% positive cells for 2; 51–75% positive cells for 3 and >76% positive cells for 4) and staining intensity (no staining = 0; slight staining = 1; moderate staining = 2; strong staining = 3). Scores for both intensity and percentage of positive cells were summed for an overall staining score. The difference in the levels of expression of PSA depending on the patient’s allele ([T] vs [C]) for the rs17632542 SNP were then analysed.

### Study populations and genotyping

The rs17632542 SNP was genotyped on the Illumina OncoArray SNP-chip^16^ in 49,941 PCa cases and 32,001 disease-free controls. Detailed information on the studies involved has been provided previously^13^. The OncoArray Consortium, a large collaborative effort to gain new insight into the genetic architecture underlying breast, ovarian, prostate, colorectal and lung cancers, developed a custom high-density genotyping array, the “OncoArray”, that included 310,000 SNPs for meta-analyses and fine-mapping for the above five cancers. Further, 80,000 PCa specific genetic markers derived from previous multi-ethnic meta-analysis^71^ (including ancestral populations of Europeans, African Americans, Japanese, and Latin Americans), fine-mapping of known PCa loci, and candidate nominations were included on the OncoArray. Briefly, 42 studies provided core data on disease status, age at diagnosis (observation or questionnaire for controls), family history, and clinical factors for cases (*e.g*. PSA at diagnosis, Gleason score, etc.) for 49,941 PCa cases and 32,001 disease-free controls. Previous GWAS contributed an additional 32,255 PCa cases and 33,202 disease-free controls of European ancestry for the overall meta-analysis^71^. For survival analysis, 37,316 cases with follow-up on cause-specific death were included. Of these, 4,629 died of PCa, 3,456 died of other causes. Cases by rs17632542 carrier status were TT= 33,281, CT= 3,909 and CC= 126.

Demographic and clinical information on the above study participants including age at diagnosis, Gleason score, stage of disease, PSA levels and cause of death were obtained through in-person interviews or medical or death records. Each study was approved by each institutional review board (IRB) and informed consent was obtained from each participant. Patient studies were conducted in accordance with the Declaration of Helsinki. Low risk disease was defined as Gleason score ≤6, PSA<10; intermediate risk as Gleason score=7 or PSA=10–20; and high-risk aggressive disease was defined as Gleason score ≥ 8 or PSA>20. Genotypes were called using Illumina’s proprietary GenCall algorithm. Serum tPSA and f/t PSA analysis were reported for 969 PCa patients (CC (2) and CT (97) genotypes compared to TT (870)).

VIP is an ongoing population-based cohort study initiated in 1986 for 43,692 men with more than 20 years of follow-up and includes residents of Västerbotten County, Sweden. A nested case-control design with three controls matched to each index case were available which included 1,743 men with a PCa diagnosis. Of these, there were 126 patients with metastatic PCa during follow-up who subsequently died from PCa^72^. Additional PCa cases (n=1,223) were available through the Malmö Diet and Cancer (MDC) cohort, a large prospective, population-based study with more than 20-years of follow-up^72^. In this cohort, 1053 cases with available mortality information were used for survival analysis^73^. Control serum samples with tPSA and f/t PSA analysis were available from the MDC (n=2,458) and the VIP (n=4,810) cohorts. Serum f/t PSA values have already been reported for these two cohorts (both cases and controls)^27^. The genotype data for the rs17632542 SNP and tPSA and fPSA levels for the MDC and VIP cohorts was available through previous GWAS^18,27^.

### Statistical analyses

Association between the rs17632542 SNP and PCa risk was analysed using the per-allele trend test, adjusted for study relevant covariates using logistic regression and seven principal components derived from analysis of the whole iCOGS and OncoArray dataset. Odds ratios (OR) and 95% confidence intervals (95% CI) were derived using SNPTEST (https://mathgen.stats.ox.ac.uk/genetics_software/snptest/snptest.html) or an in-house C++ program. Tests of homogeneity of the ORs across strata were assessed using a likelihood ratio test. The associations between SNP genotypes and PSA level were assessed using linear regression, after log-transformation of PSA levels to correct for skewness. In a case-only analyses, Cox proportional hazards regression was used to estimate associations of each SNP. To assess the association between the *KLK3* c.536T>C variant and prognosis after a PCa diagnosis, we used time to event analysis with the primary end point being death from PCa or other causes. Survival time was calculated from the date of diagnosis until the date of death from PCa or all causes other than PCa or, if still alive, the date at last follow-up. Survival analyses were limited to cohorts for which follow-up for cases was at least 90% complete and that have at least 5 PCa deaths. A total of 37,316 men with PCa were used in this analysis (cases by carrier status, TT= 33,281, CT= 3,909 and CC= 126). All regression analyses were performed using SPSS, R and Stata 14^13^. To address the effect of the SNP on f/t PSA levels, all models included study site and principal components as covariates. The associations between SNP genotypes and PSA levels were assessed using linear regression in R, adjusted for age of the subject at the time of blood draw. The tPSA and fPSA values were log-transformed to limit potential bias because of deviation from normality. All statistical tests were two-sided.

For *in-vivo* intracardiac models (n=7/group), two mice in Thr^163^ PSA group and one mouse in vector group died due to unrelated bacterial infection and were excluded. Unless otherwise stated, for all other biological or biochemical analyses three independent experiments were conducted with results presented as mean +/− standard deviation, and analyzed using a Kruskal-Wallis test, Student T-test, one-way ANOVA or two-way ANOVA with a *p*-value of <0.05 considered statistically significant.

## Supplementary Material

1
