# Supplementary material for: Biochemical activity induced by a germline variation in KLK3 (PSA) associates with cellular function and clinical outcome in prostate cancer
Source: Res Sq. 2023 Mar 28:rs.3.rs-2650312. Preprint. [Version 1] doi: 10.21203/rs.3.rs-2650312/v1 (PMC10081352; doi:10.21203/rs.3.rs-2650312/v1)
Supplement: 1 [file NIHPPRS2650312V1-supplement-1.pdf]

Supplemental Information

Supplementary Table 1. Expression of PSA from recombinant engineered PSA constructs quantified by DELFIA PROSTATUS immunoassay

Supplementary Table 2. Participant characteristics

Supplementary Table 3. Risk of prostate cancer for rs17632542 SNP

Supplementary Table 4. Frequency distribution for the rs17632542 SNP

Supplementary Table 5. Primers used for the study

Supplementary Figure 1. *KLK3* expression in overexpression models
Representative mRNA analysis demonstrating the expression of PSA in PSA transfected PC-3 and MSK3 clones: Wt PSA, Thr^{163} PSA and inactive mutant Ala^{195} PSA. Results are shown as the mean ± SEM from two qRT-PCR experiments, each with three replicates.

Supplementary Figure 2. Digital spheroid analysis of PC-3 and MSK3 cells
A) A gray channel image generated from the original image was corrected to reduce the background. A density image was generated for the detection of spheroids as cell agglomerations with high cell numbers per area and their separation from isolated cells distributed across the wells. Positive objects (confirmed spheroids) were split into three areas, with the green contour indicating the outer core, the orange contour labelling the inner core and the blue contour highlighting the regions with detectable cells in the periphery. Quantitative analyses for the area and circularity of PC-3 spheroids were determined by the StrataQuest^{TM} software. **B)** Dead (red) and live (green) cells count and mean intensity within the spheroid was detected based on setting thresholds, spheroid number, and event area for spheroid area were measured for the MSK3 spheroids. **C)** Number of MSK3 spheroids in selected field (average number from two images). See also Figure 2A–2G

Supplementary Figure 3. Confocal Microscopy for PSA variants and vector transfected PC-3 cells on OBM constructs
A) Shape Factor of PC-3 cells to OBM constructs after 12 h co-culture. **B)** Confocal laser microscopy images from PC-3/OBM constructs after 1 day and 10 days co-culture showing, from left to right, a volume snapshot of all channels and the maximum projections of z-stacks (mKO2 (red) for PC-3, GFP (green) for Phalloidin, and DAPI channel (blue) showing nuclei of both cancer cells and osteoblasts. For **A-B**, 2 technical replicates were used, 4-5 fields of view/replicate, for a total of 120-230 cells per condition. *P* values on all groups were evaluated by one-way ANOVA followed by Games-Howell post hoc analysis. See also Figure 2H-2J.

Supplementary Figure 4. Effect of rs17632542 SNP on PC-3 cell metastasis in an experimental metastasis mice model
A) Representative photographs of resected liver and kidneys from mice following cardiac injection of PC-3-Wt/Thr^{163} PSA (n=7 mice/group). Increased tumour lesions are observed in the livers of Thr^{163} PSA injected mice. **B)** X-ray images of tumour-bearing hind legs of mice; red areas indicate areas of bone degradation, suggesting presence of tumour. **C**) H&E staining of tumour-bearing hind leg bones. **D**) Representative bioluminescence images of tumour-bearing mandibles of mice (week 4) post cardiac inoculation. **E**) Scatter plots of tumour bioluminescence based on region of interest (ROI) drawn over the jaw; horizontal line indicates

median value. Statistical analysis was Dunn's multiple comparisons test. **F**) Mean bioluminescence values from ROI drawn over entire animals from each group, over multiple weeks. **G**) Serum concentration of total PSA at endpoint from mice injected intracardiac with tumour cells. Statistical analysis was Mann Whitney test. **H**) *In-vitro* bioluminescence images of cell lines seeded by 2-fold serial dilution, starting at 50,000 cells per well. Also see Figure 2K-2O

Supplementary Figure 5. Proteolysis of full-length protein substrates by mature PSA protein variants

A) Casein zymography of Wt PSA and Thr^{163} PSA: One µg of Wt PSA, Thr^{163} PSA and inactive mutant Ala^{195} PSA (from left to right) were resolved on a 10% casein zymogram Protein Gel (Invitrogen) followed by Coomassie brilliant blue R-250 (0.25% w/v) staining. Clear zones due to protease activity were observed in the Wt PSA and Thr^{163} PSA lanes only. The bottom gel represents the silver stain analysis to indicate equal protein loaded into the wells. **B)** Michaelis-Menten kinetics for PSA protein variants: Michaelis-Menten kinetic analysis of Wt PSA (red), Thr^{163} PSA (blue) and inactive mutant Ala^{195} PSA (grey) for two substrates MeO-Suc-RPY-AMC and Mu-HSSKLQ-MCA. Kcat values showed the Thr^{163} PSA protein variant had decreased substrate activity in comparison to Wt PSA (mean ± SEM; n=3). Also see legend to Figure 3B. (**C)** Silver stain analysis of mature PSA variants (0.2 µM) incubated for 22 h with full-length substrates (semenogelin-1, galectin-3, fibronectin, nidogen-1, and laminin α-4) (0.5 µM) at 37°C, indicated that the Thr^{163} PSA isoform exhibited lower proteolytic activity compared to the wild type (Wt) PSA. Ala^{195} PSA had less effect. Wt PSA efficiently cleaved full-length fibronectin and laminin α-4, while partial proteolysis was observed with nidogen-1. The full-length proteins (orange arrow), PSA band (blue arrow) and their corresponding molecular weights are indicated. Cleaved products of the substrates (green arrows) due to PSA proteolytic activity are indicated to the right. High molecular weight bands that may correspond to the dimers of the full-length protein or their aggregates were observed above their expected size bands. Molecular weight of the protein standard (kDa) is indicated to the left. Also see Figure 3C-D. **D)** HUVECs treated with different recombinant PSA protein variants (250 nM) (Wt, Thr^{163} and Ala^{195} PSA) and the graph to the right represents the angiogenesis index. Thr^{163} PSA exhibited lower anti-angiogenic potential compared to Wt PSA (n=2, mean ± SEM, **P*<0.01 as compared to control (t-test)). Scale bar is 500 µm. Also see Figure 3F

Supplementary Figure 6. Overall- and metastasis-free survival of MDC and VIP cohorts for the rs17632542 SNP

A-B) Overall survival as measured by cumulative incidence of death from PCa for the rs17632542 SNP in **A)** MDC (n=1,053), HR= 1.39, 95% CI=0.98-1.98, *P*=0.06; and **B)** VIP cohorts (n=1,644), HR=1.69, 95% CI=1.07-2.65, *P*=0.03. **(C)** Metastasis free survival analysis estimated by Kaplan Maier plot in the VIP cohort of 1,381 prostate cancer cases. rs17632542 is associated with metastasis-free survival time in VIP cohort (HR=1.65, 95% CI=1.03-2.62, P=0.05).
